# Roles of Erythroid Differentiation Regulator 1 (Erdr1) on Inflammatory Skin Diseases

**DOI:** 10.3390/ijms17122059

**Published:** 2016-12-08

**Authors:** Youn Kyung Houh, Kyung Eun Kim, Hyun Jeong Park, Daeho Cho

**Affiliations:** 1 Department of Biological Sciences, Sookmyung Women’s University, Seoul 04310, Korea; bobada6025@gmail.com; 2 Department of Cosmetic Sciences, Sookmyung Women’s University, Seoul 04310, Korea; kyungeun@sookmyung.ac.kr; 3 Department of Dermatology, Yeouido St. Mary’s Hospital, The Catholic University of Korea, Seoul 07345, Korea

**Keywords:** Erdr1, IL-18, cutaneous inflammation, melanoma, psoriasis, rosacea

## Abstract

Erythroid Differentiation Regulator 1 (Erdr1) is known as a hemoglobin synthesis factor which also regulates cell survival under conditions of stress. In addition, previous studies have revealed the effects of Erdr1 on cancer progression and its negative correlation with interleukin (IL)-18, a pro-inflammatory cytokine. Based on this evidence, the therapeutic effects of Erdr1 have been demonstrated in several inflammatory skin diseases such as malignant skin cancer, psoriasis, and rosacea. This article reviews the roles of Erdr1 in skin inflammation, suggesting that Erdr1 is a potential therapeutic molecule on inflammatory disorders.

## 1. Introduction

Erythroid Differentiation Regulator 1 (Erdr1) is a recently identified cytokine that was first discovered as a hemoglobin synthesis factor in human and murine erythroleukemia cell lines. Expression of Erdr1, a highly conserved autocrine factor, is widely distributed across many normal mouse and human tissues [1]. Erdr1 is localized at the inner part of cytoplasmic membrane and is secreted through vesicles. Studies have also revealed Erdr1 as a stress-related factor from stroma which controls the survival and growth of hematopoietic progenitors [2]. Erdr1 is secreted immediately from cells in response to several stress conditions. Erdr1 enhances cell survival at low concentration and low cell density but increases cell death at high concentration and high cell density. These opposing effects of Erdr1 on cells are essential for maintaining growth homeostasis.

Recently, Erdr1 has been demonstrated to play additional functions in cancer. This protein regulates the activity of several cancers by inhibiting cell motility and growth. In murine melanoma, Erdr1 overexpression suppressed metastasis in the lung in vivo and inhibited migration, invasion, and proliferation in vitro [3]. Gastric cancer frequently metastasizes to other parts of the body, making it one of the most fatal cancers [4]. Exogenous treatment with recombinant Erdr1 significantly inhibited the migration and invasiveness of gastric cancer by inducing *E*-cadherin [5]. Additionally, Erdr1 can indirectly repress cancer activity by activating the immune system, specifically enhancing natural killer (NK) cell activation [6]. NK cells are effector lymphocytes which can eliminate tumor cells through cytolytic granule exocytosis and induction of target apoptosis [7]. During NK cell-mediated target cell killing, apoptosis-inducing enzymes such as perforin and granzymes are localized to the tumor in the immunological synapse formed between both cells [8]. Lee et al. reported that exogenous treatment of recombinant Erdr1 enhanced the effect of primary human NK cell cytotoxicity of leukemia cancer cells [6]. By regulating actin rearrangement, Erdr1 increases the accumulation of F-actin at the immunological synapse of NK cells. As a result, NK cell cytotoxicity against the target cancer is enhanced. Taken together, Erdr1 can repress cancer progression through inhibition of cancer cell motility and activation of the immune response.

Jung et al. reported that Erdr1 expression is negatively regulated by interleukin (IL)-18, a pro-inflammatory cytokine, in a murine melanoma cell line [3]. In the study, knockdown of IL-18 in B16F10 cells led to increased Erdr1 expression at the RNA and protein levels. The negative correlation between Erdr1 and IL-18 was also confirmed in a human gastric cancer cell line [5]. Both of these cell types have been reported to exhibit a high level of IL-18 expression that is related to cancer progression, including proliferation, metastasis, and angiogenesis [9,10]. However, Erdr1 produces the opposite effect on IL-18 in melanoma and gastric cancer. Due to its role as a pro-inflammatory cytokine, high levels of IL-18 are detected in diseases associated with chronic inflammation [11,12]. Moreover, IL-18 is expressed not only by immune cells but also by non-immune cells like keratinocytes and epithelial cells. In particular, IL-18 plays an important role in cutaneous inflammation [13,14]. Therefore, suppression of IL-18 bioactivity can be an effective therapeutic approach for treating chronic inflammatory skin diseases. Accordingly, much research involving Erdr1 has been focused on inflammatory diseases on account of its negative correlation with IL-18.

## 2. Roles of Erythroid Differentiation Regulator 1 (Erdr1) on Inflammatory Skin Diseases

Skin is the largest frontline host defensive tissue against pathogens and consists of two layers. The outer protective barrier, called the epidermis, is composed of keratinocytes, melanocytes, langerhans cells, and merkel cells [15]. The dermis, or lower layer, includes immune cells such as CD4^+^ T cells, CD8^+^ T cells, and macrophages for execution of the immune response upon pathogen invasion [16,17].

As part of its protective function against chemical and physical insults, skin is involved in the innate immune response. Inflammation is one of the key responses of the innate immune system which, along with the adaptive immune response, comprises a complex biological reaction [18]. Injury, infection, and irritation can trigger inflammation in skin tissues, resulting in such common symptoms as heat, pain, redness, and swelling. The purpose of inflammation is to eliminate the initial causes or damaged cells and begin tissue repair. However, long-term inflammation caused by genetic or environmental factors can lead to chronic cutaneous diseases such as psoriasis, rosacea, atopic dermatitis, and alopecia areata, which are associated with uncontrolled immune activity [19]. A variety of cytokines play an important role in the inflammatory pathogenesis of skin. Cutaneous and systemic overexpression of several inflammatory cytokines such as IL-1, IL-6, IL-8, IL-12, IL-18, interferon (IFN)-γ, and tumor necrosis factor (TNF)-α has been demonstrated [20,21].

It has been reported that uncontrolled production and activation of IL-18 in skin contributes to the pathogenesis of chronic inflammatory diseases [22]. In fact, increased expression of IL-18 and its receptor were observed on lesional skin tissues from patients diagnosed with inflammatory cutaneous diseases such as psoriasis, rosacea, atopic dermatitis, or alopecia areata [19]. Moreover, plasma IL-18 concentration has been linked to the severity of these diseases. Therefore, IL-18 has been considered as a possible marker of various inflammatory skin disorders [23,24,25,26].

Erdr1, an IL-18-regulated novel factor, is also expressed in normal skin cells, including keratinocytes and melanocytes [3]. Previous reports revealed that Erdr1 may possibly be associated with skin abnormalities. Kim et al. suggest that Erdr1 is a pro-apoptotic factor in human keratinocytes [27]. Expression of Erdr1 was increased in the HaCaT cell line following ultraviolet B (UVB) irradiation and exogenous treatment of recombinant Erdr1 enhanced UVB-induced apoptosis via caspase-3 activation.

Here, we review recent findings that highlight the correlation between Erdr1, an IL-18-regulated factor, and chronic inflammation in cutaneous disorders such as melanoma, psoriasis, and rosacea.

### 2.1. Effects of Erdr1 on Malignant Skin Cancer and Melanoma

Melanoma is one type of malignant skin tumor that arises from melanocytes localized in the epidermal skin layer [28]. Chronic inflammation has been suggested as one of the hallmarks of exposure to ultraviolet (UV) radiation and other environmental factors that cause skin cancers [29]. Recently, much molecular evidence that is focused primarily on the role of inflammation in melanoma has been accumulating. These data include the role of TNF, IL-1, IL-6, IL-18, matrix metalloproteases (MMP), and vascular endothelial growth factor (VEGF), which are NF-κB-regulated inflammatory factors predominantly expressed in cancer. In addition, the activity of NF-κB is usually regulated by other transcription factors such as signal transducer and activator of transcription 3 (STAT3) [30].

Jung et al. found that Erdr1 expression was higher in normal melanocytes compared with melanoma cell lines. This result was also confirmed in human skin tissues from melanoma patients and healthy donors, indicating that Erdr1 expression is downregulated in melanoma. In the study, Erdr1 expression was found to be negatively regulated by IL-18, which has a pro-cancer effect on melanoma [3]. Overexpression of Erdr1 significantly inhibited cell migration, invasion, and proliferation in B16F10, a murine melanoma, in vitro. In addition, melanoma lung metastasis and tumor growth were suppressed in mice implanted with Erdr1-overexpressing melanoma. This study found that Erdr1 overexpression leads to the downregulation of heat shock protein 90 (HSP90), a ubiquitous chaperone that reportedly acts as a pro-cancer factor which is enhanced in advanced malignant melanoma related to stress conditions. Studies have established HSP90 as a marker of progression in melanoma and a therapeutic target in cancer [31,32]. Inhibition of HSP90 suggests that Erdr1 acts as a regulatory protein for melanoma motility. Moreover, HSP90 regulates NF-κB signaling, an important pathway involved in mediating the inflammatory response [33]. In fact, HSP90 inhibitors have been shown to attenuate inflammatory responses, suggesting that HSP90 could be a therapeutic target for treating various inflammatory skin disorders [34,35].

Melanoma is resistant to diverse conventional chemotherapy due to its aggressiveness [36]. Therefore, development of an effective therapeutic strategy involving apoptosis of cancer cells is necessary. Lee et al. showed that Erdr1 could suppress murine melanoma growth through the regulation of apoptosis in vitro and in vivo [37]. Intraperitoneal injection of recombinant Erdr1 significantly reduced melanoma growth implanted onto mouse skin. Decreased B-cell lymphoma 2 (Bcl-2) and increased bcl-2 like protein 4 (Bax) expression were detected on tumor tissues from the affected mouse. Moreover, exogenous treatment of recombinant Erdr1 on B16F10 cells increased apoptosis by reducing Bcl-2 expression while enhancing Bax in vitro. High expression of Bcl-2, an anti-apoptotic factor, has been associated with resistance to chemotherapy in human melanoma and other tumor types [38]. Another Bcl-2 family member, Bax, exhibits pro-apoptotic effects, thereby opposing Bcl-2. Clinical trials for treating melanoma that target the Bcl-2 family are important because these proteins act as regulators of the mitochondrial apoptotic pathway [39,40]. Activity of the well-established apoptosis-related signaling molecule STAT3 was also inhibited by exogenous Erdr1 [41]. As mentioned, activation of STAT3 mediates tumor-promoting inflammation and can regulate the critical transcription factor, NF-κB. Thus, downregulation of STAT3 activity has been considered a promising strategy for treating inflammatory diseases and cancer [42].

The relationship between melanoma and IL-18 has been widely studied. Studies have demonstrated that IL-18 enhances the migration of murine melanoma cells via the generation of “reactive oxygen species” (ROS) and the MAPK pathway [43]. Metastatic patients showed significantly higher IL-18 expression than both healthy controls and non-metastatic patients [44,45]. Therefore, an IL-18R-expressing phenotype may be a diagnostic biomarker indicating predisposition of melanoma metastasis [45,46]. Melanomas developed through inflammation-dependent mechanisms have high metastatic potential that is dependent on IL-18, which can activate the STAT3 pathway.

**Figure 1:**
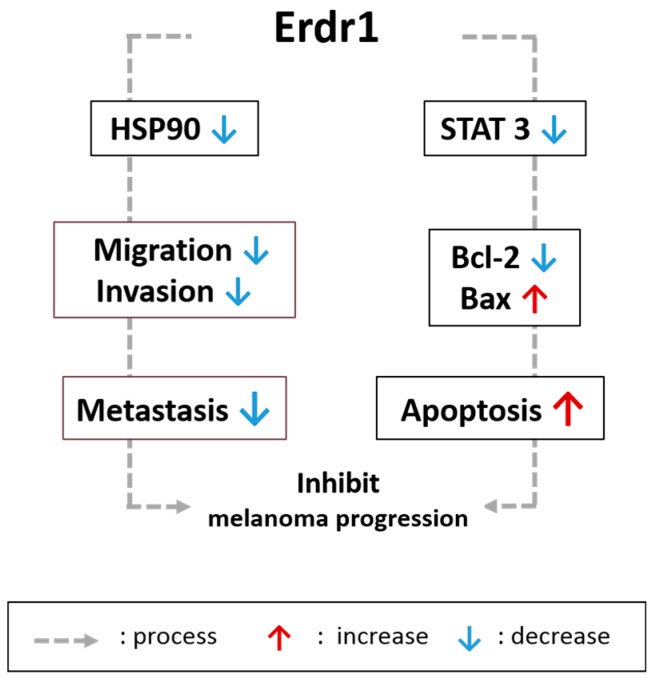
Erdr1 inhibits melanoma progression. Erdr1 suppresses metastasis by downregulating migration and invasion ability via HSP90 inhibition. In addition, Erdr1 reduces the apoptosis-regulating STAT3 signaling pathway. Erdr1 exerts its anti-cancerous effects by regulating HSP90 and STAT3 in melanoma.

Taken together, the negative correlation between Erdr1 and IL-18 suggests a potential anti-inflammatory effect that occurs through regulation of inflammatory response-related molecules such as HSP90 and STAT3, which are also candidates to be targeted for melanoma therapy (Figure 1).

### 2.2. Roles of Erdr1 on Psoriasis

Psoriasis is a common chronic skin disease that affects 2%–3% of the global population and is considered an immune-mediated inflammatory disorder. Psoriatic skin lesions are characterized by the manifestation of red and white scaly plaques on the top layer [47]. Environmental (stress, microorganisms, drugs, trauma, smoking, etc.) and genetic factors initiate stressor (LL-37) production from epidermal keratinocytes [48,49]. They activate myeloid dendritic cells to induce the differentiation of naïve T cells into effector cells such as Th1 and Th17 cells [50]. These effector cells expressing the specific chemokine receptors escape from blood vessels and migrate into skin tissue along chemokine gradients. Then, the cells secrete several pro-inflammatory cytokines, such as interferon-γ, TNF-α, IL-17, and IL-22, for keratinocytes to respond. Recent research has been focused on IL-17-producing Th17 cells. Therefore, this response is unique to epithelial immunity which bridges the adaptive immune response and keratinocyte dysregulation in psoriasis [51].

Erdr1 has been suggested to act as a stress-related factor that regulates cell growth and survival [2]. Kim et al. first reported that Erdr1 may be associated with skin abnormalities [27]. In the skin, UVB irradiation is a common stressful condition which causes DNA damage and leads to apoptosis [52,53]. Enhancement of both Erdr1 mRNA and protein expression were enhanced by UVB irradiation depending on Erk and p38 MAPK phosphorylation in HaCaT, a human keratinocyte cell line [54]. Moreover, UVB-mediated apoptosis was reduced in Erdr1 knockdown cells whereas apoptosis was increased in Erdr1-overexpressing cells. These data demonstrate that apoptosis induced by UVB is dependent on Erdr1 expression in keratinocytes. UVB irradiation is a very harmful biological stressor, so a better outcome for cells is to undergo suicide via apoptosis rather than permanent mutation. Therefore, as a pro-apoptotic factor, Erdr1 could be a therapeutic molecule for treating skin diseases, such as skin cancer and psoriasis, which are characterized by low apoptosis and uncontrolled cell proliferation.

**Figure 2:**
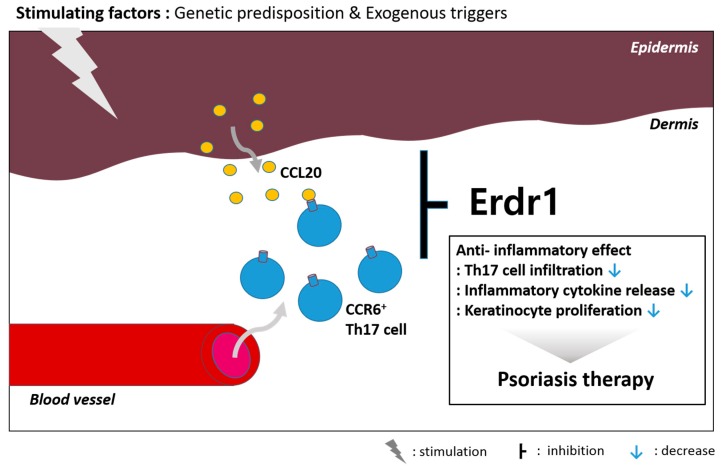
Erdr1 is a potential therapeutic molecule for psoriasis due to its role in suppressing inflammation. Genetic and environmental triggers stimulate the epidermis, which produces chemokines for inflammatory immune cell infiltration. More specifically, Erdr1 inhibits the infiltration of CCR6^+^ Th17 cells via blockade of CCL20.

Keratinocytes stimulated by various genetic predispositions or exogenous triggers exhibit hyper-proliferation and aberrant differentiation, releasing antimicrobial peptides as well as chemokines and inflammatory cytokines including IL-18 [55,56,57]. In fact, IL-18 levels are significantly higher in skin lesions of psoriasis patients relative to healthy controls [58]. Because serum IL-18 concentration is linked to psoriasis severity (PASI), IL-18 might be considered as a possible biomarker of psoriasis [24]. In HaCaT cells, Erdr1 expression is also inversely correlated with IL-18 [59]. Kim et al. compared Erdr1 expression in skin tissues from human psoriasis patients and healthy volunteers. Histological analysis by immunohistochemistry staining suggested that Erdr1 was significantly downregulated in psoriatic lesional skin compared to healthy skin. Meanwhile, IL-18 expression exhibited the opposite expression pattern. Moreover, after effective therapeutic treatment using cyclosporine A in a psoriasis-like mouse model, the reduced Erdr1 mediated by induction of psoriasis-like skin inflammation was reversed and symptoms were relieved [60]. Intraperitoneal administration of recombinant Erdr1 exerted a therapeutic effect on a psoriasis-like mouse model which was induced by imiquimod via activating the IL-23/IL-17 axis [61]. Erdr1 significantly improved redness, thickness and prevalence of scales of lesional skin of a psoriasis-like skin inflammatory mouse model. Furthermore, keratinocyte acanthosis, parakeratosis, and desquamation, as well as inflammatory infiltration, were alleviated in psoriasis-like lesions. Various biomarkers for psoriasis, such as keratins 6, 14, 16, and S100A8, were reduced by Erdr1. Major inflammatory cytokines such as IL-17, IL-22, and TNF-α are also reduced by Erdr1 administration. Additionally, Erdr1 administration inhibited CCR6^+^ cell infiltration into epidermal sites by blocking the production of CCL20, a chemokine for CCR6^+^ cells, from keratinocytes [60]. In psoriasis pathogenesis, CCR6^+^ Th17 cells are highly accumulated in lesional skin, stimulating keratinocytes through IL-17 production [62]. Therefore, the result suggest that Erdr1 reduces CCR6^+^ Th17 migration toward the lesional site. Taken together, Erdr1 may be involved in psoriasis pathogenesis, making it a potential biomarker and therapeutic molecule (Figure 2).

### 2.3. Therapeutinc Effect of Erdr1 on Rosacea

Besides psoriasis, rosacea is a common chronic inflammatory skin disease which mainly affects the central facial skin tissue, including the forehead, nose, chin, and cheeks [63]. The pathology of this disease results from very complex communication between the cutaneous vascular, nervous, and immune system in the skin [63,64,65]. Angiogenesis is a characteristic of rosacea and plays a critical role in the inflammatory response, leading to exacerbation of rosacea pathogenesis. Angiogenesis is vital for many physiological and pathological reactions, including wound healing, inflammation, tumor growth, and metastatic spread. In particular, VEGF, which is known as a major skin angiogenesis factor, is released by epidermal keratinocytes on lesional rosacea tissues [66].

Trigger factors such as LL-37 stimulate the innate immune response and cause neurovascular dysfunction, leading to vasodilation, inflammation, fibrosis, and neurosensory system activation. An altered innate immune response results in both inflammation and angiogenesis [67]. In rosacea skin, perivascular infiltration and inflammation can be observed throughout the upper to deep dermis. Erdr1 expression has been examined in the skin tissues of patients with rosacea [68]. Histological analysis revealed that Erdr1 expression is lower in patients with rosacea than normal controls. However, IL-18 exhibited a higher expression level in rosacea patients compared to healthy donors.

**Figure 3:**
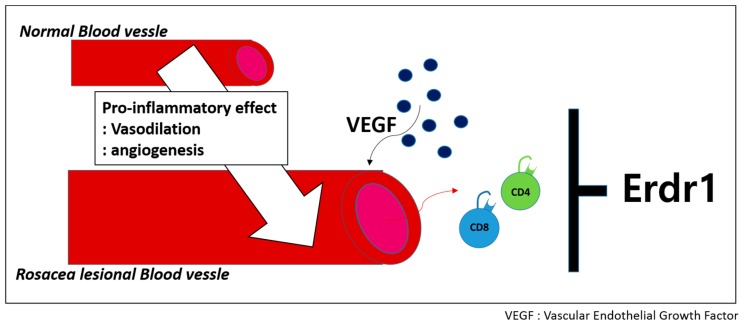
Erdr1 is suggested as a candidate target for rosacea treatment due to its anti-inflammatory and anti-angiogenic effects.

In addition, a therapeutic effect of Erdr1 on a rosacea-like mouse model involving the regulation of angiogenesis was identified. Intraperitoneal administration of recombinant Erdr1 relieved clinical rosacea symptoms in an LL-37-induced rosacea mouse model. Decreased VEGF expression and suppressed CD34^+^ microvessels were also detected on the skin tissues of Erdr1-treated animals, suggesting that Erdr1 suppresses angiogenesis. Additionally, an anti-inflammatory effect of Erdr1 mediated by reduced infiltration of inflammatory cells such as CD4^+^ and CD8^+^ T cells was demonstrated. These data suggest that Erdr1 suppresses T cell-mediated inflammation (Figure 3).

Studies have suggested that IL-18 is an angiogenic mediator due to its ability to induce endothelial tube formation in vitro and in vivo [69]. Enhanced expression of VEGF family members and their receptors was detected in the skin of rosacea patients, suggesting that VEGF is a potential therapeutic target through inhibiting angiogenesis [67,70]. Several therapeutic agents which decrease inflammation and control angiogenesis via inhibition of VEGF production and neovessel formation have been introduced to treat rosacea [71,72]. Overall, these data suggest that Erdr1 can be an effective therapeutic candidate via its anti-inflammatory and anti-angiogenic effects.

## 3. Conclusions

This article reviews the effects of Erdr1 on inflammatory skin diseases. It has been reported that Erdr1 overexpression suppressed melanoma metastasis by reducing HSP90 expression. HSP90 has a pro-inflammatory effect as well as a pro-cancerous one, suggesting that HSP90 could be a therapeutic target for treating inflammatory diseases, especially in psoriasis. In fact, HSP90 inhibitor alleviated psoriasis-like skin inflammation in a mouse model [35]. Additionally, recombinant Erdr1 treatment reduced STAT3 activity in melanoma cell line. STAT3 is known as a critical signaling molecule involved in cancer and inflammation. Epidermal keratinocytes in psoriatic lesions are characterized by activated STAT3. STAT3 inhibitors markedly improved psoriasis symptoms in patients [73]. Taken together, Erdr1 exerts a therapeutic effect on the inflammatory skin diseases due to its overall anti-inflammatory effects. Besides skin diseases, Erdr1 can be a potential therapeutic molecule for other disorders that are characterized by inflammation.
